# Green Pretreatment of Tropical Fruit Peels Using Triethylammonium Hydrogen Sulfate: A Route Toward Sustainable Biomass Valorization

**DOI:** 10.1021/acsomega.5c10185

**Published:** 2026-02-02

**Authors:** Leonardo A F Souza, Crystian Ribas, Irede Dalmolin, Marcelo Bortoli, Tania Maria Cassol

**Affiliations:** † School of Chemical Engineering (FEQ), Universidade Estadual de Campinas (Unicamp), Av. Albert Einstein, 500, Cidade Universitária, Campinas, SP 13083-852, Brazil; ‡ Universidade Tecnológica Federal do Paraná (UTFPR), R. Gelindo João Folador, 2000, B. Novo Horizonte, Francisco Beltrão, PR 85602-863, Brazil

## Abstract

The search for biofuels has recently intensified because of the urgent need to replace fossil fuels with renewable alternatives. Investigation of biomass, especially waste, presents an excellent option for biofuel production, including second-generation (2G) ethanol, which can be produced from lignocellulosic waste. 2G Ethanol production requires pretreatment and hydrolysis of biomass to break down cellulose, generate higher amounts of sugars, and consequently increase production yields. Ionic liquids (ILs), composed of organic and inorganic ions, have low melting points, low vapor pressures, and the ability to solubilize cellulose, making them effective in breaking down cellulose and thus emerging as an efficient alternative to acid pretreatment. Therefore, this study aimed to evaluate the efficiency of triethylammonium hydrogen sulfate IL in the pretreatment of tropical fruit peel residues such as bananas, oranges, and mangoes. For this purpose, the biomass was characterized through sugar quantification and determination of ash, moisture, extractives, holocellulose, α-cellulose, and hemicellulose content. Two pretreatment processes were conducted for lignocellulosic biomass: one in an oil bath and the other in an oven. Additionally, yield analyses, scanning electron microscopy (SEM), and infrared spectroscopy (IR) were performed on the products obtained from the pretreatments. Based on characterization analyses of the raw materials, mango residue was identified as the biomass with the highest potential for bioethanol production, followed by orange and banana residues owing to its high sugar content, low ash and moisture content, and favorable cellulosic composition. Among the evaluated pretreatments, the oven method showed the best results in weakening the lignin-hemicellulose-cellulose complex and lignin precipitation, also indicating mango residues as being the most promising in terms of cellulose pulp production and lignin removal. This study adds value by demonstrating a low-cost and practical approach to pretreating abundant tropical fruit peel residues using triethylammonium hydrogen sulfate, an accessible and more affordable ionic liquid. Additionally, it provides significant scientific value by addressing two major global challenges: the need for renewable energy alternatives and the growing demand for sustainable waste valorization methods.

## Graphical abstract

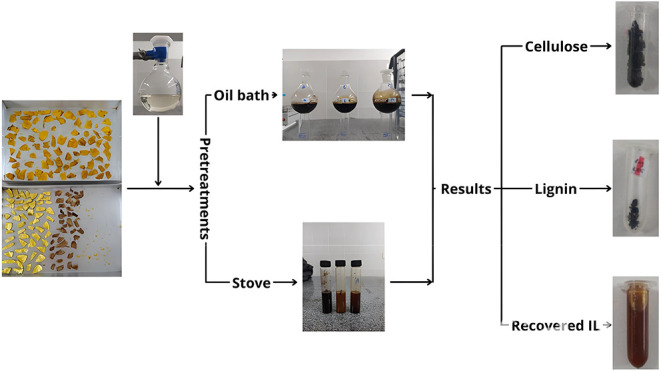

## Introduction

The oil crisis of the 1970s spurred the search for alternatives to fossil fuels, which was further exacerbated by growing concerns about greenhouse gas emissions and their climate impacts.^1^

Biomass rich in lignocellulosic materials has been gaining prominence as a raw material for many biofuels, including bioethanol, biogas, bio-oil, charcoal, nanocellulose, bioactive compounds, and chemicals.^2,3^ Pretreatment is a crucial step in enhancing the efficiency of biofuel production because it disrupts the lignin-hemicellulose-cellulose complex and exposes cellulose for conversion into fermentable sugars and other products. Various methods can be employed for this purpose, including mechanical, biological, chemical, physical, and physicochemical processes.^4^ Ionic liquids (ILs) have emerged as promising alternatives to chemical pretreatment.^5,6^ These compounds are composed of organic and inorganic ions and have low melting points, low vapor pressures, and high cellulose solubilization capacity.

ILs have also gained attention as green solvents and catalysts for organic reactions, proving to be a viable route for cellulose dissolution.^7–9^ Moreover, they can be tailored to suit the specific requirements of each application, thereby enhancing the versatility. Additionally, due to their low environmental impact during production and the possibility of recycling and reuse, the application of ILs helps reduce biofuel production costs. This promotes eco-friendly characteristics.^10^ However, technical and economic challenges must be overcome to enable large-scale implementation.^11,12^

Triethylammonium Hydrogen Sulfate^13^ (Figure 1) is a Brønsted Acidic IL. Its synthesis consists of an acid–base reaction, adding sulfuric acid to triethylamine. This IL arise to be very promising for the pretreatment of biomass, principally because it is a very cheap when compared to others.^14^

**Figure 1:**
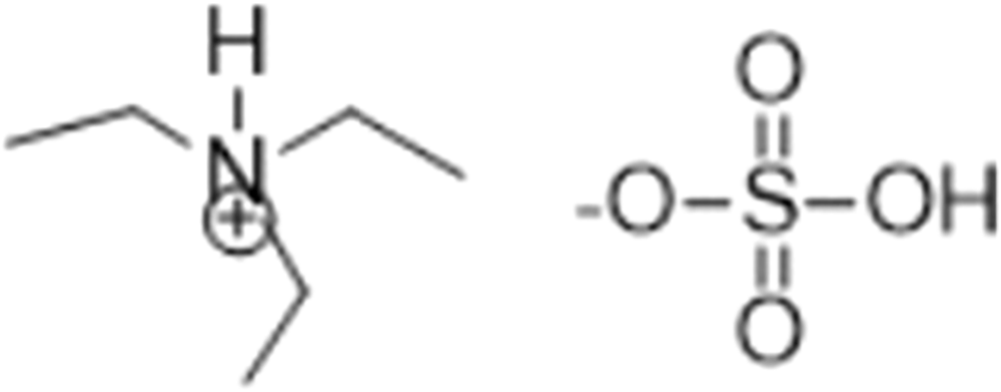
Triethylammonium hydrogen sulfate ionic liquid.

In 2018, Gschwend et al.^15^ reported favorable outcomes when employing Triethylammonium Hydrogen Sulfate as a rapid pretreatment in the dissolution of lignocellulosic material from the species Miscanthus at elevated temperatures. Similarly, Najya Jabeen Poolakkalody et al.^16^ utilized the same ionic liquid for the pretreatment of the perennial grass, *Pennisetum polystachion*, in the production of biofuels. Other researchers have also employed this ionic liquid for biomass pretreatments, such as the delignification of corn straw using ethanol as a solvent, achieving 80% removal of lignin at a temperature of 78 °C.

Brazil is the third largest fruit producer globally, following China and India, with a total production of 59 million tons.^17^ The fruits produced in Brazil are predominantly consumed fresh and are primarily utilized in its natural state, resulting in a substantial amount of waste.^18^ In the current context of the increasing demand for environmentally sustainable materials, the indiscriminate disposal of this waste is no longer acceptable. Consequently, researchers are actively investigating methods to repurpose fruit waste, including the development of novel food products, extraction of bioactive compounds, and production of biofuels.^19,20^

Studies have reported the use of tropical fruit peels, showing their potential as sources of fiber and prebiotics. It has been demonstrated that only 2% of these peels significantly increase the growth of lactic acid bacteria.^21^

Recently, Ahmad et al.^22^ reviewed the extraction and modification of cellulose from tropical fruit peels, highlighting its economic and environmental value in waste recovery. This review addresses the extraction methods and applications of cellulose derivatives, highlighting the main challenges.

In addition, fruit peels are sources of pectin and numerous bioactive compounds, such as phenolic compounds, flavonoids, carotenoids, vitamin C, and fibers, which can be added or used in functional foods, nutraceuticals, pharmaceuticals, cosmetics, and agriculture.^23^

This study aims to explore the application of tetramethylammonium hydrogen sulfate (TEA) as an ionic pretreatment agent in the dissolution of lignocellulosic material derived from organic fruit waste, specifically banana, orange, and mango. Recognizing TEA’s role in facilitating the separation process of lignocellulosic material, the research will investigate its use in two distinct pretreatment methods, highlighting a promising approach involving the application of a green solvent. This investigation assessed the versatility and applicability of TEA, thereby contributing to the development of more sustainable methods for the recovery of waste from these fruits.

## 2 Experimental Section

All chemical reagents used in this study were purchased from Sigma-Aldrich and used as received, unless otherwise noted.

### 2.1 Raw Material Preparation

Peels residues of bananas mix (Caturra variety; *Musa cavendishii* and Prata variety; *Musa sapientum*), oranges (Pera variety, *Citrus sinensis*), and mangoes (Palmer and Tommy Atkins varieties; *Mangifera indica* L.) were collected from markets in Francisco Beltrão, Paraná, Brazil. The residues were cut into smaller portions and dried in a circulating air oven (SL 102 Solab, Brazil) at 60 °C for 48 h. The dried residues were then ground in a blender and sieved through 60 mesh openings to standardize the biomass produced. These dried and grounded residues produced were denominated flour samples.

Moisture and volatile contents were determined by placing the sample in an oven (SL 102 Solab, Brazil) under forced air circulation at 105 °C until constant weight was reached. The moisture and volatile contents were expressed as a percentage based on wet weight.^24^

### 2.2 Determination of Total Sugar Content before Pretreatment

The concentration of reducing sugars was determined using the DNS (3,5-dinitrosalicylic acid) method,^25^ utilizing the dehydrated biomass prepared from banana (BP), orange (OP), and mango peels (MP).

The samples were then diluted in distilled water to 0.5 g of 100 mL. For banana and mango samples, an additional 10-fold dilution is required. After dilution, the samples were centrifuged at 3700 rpm for 30 min. Samples with a high sucrose content underwent a prior hydrolysis process. The samples were analyzed using the DNS method, and absorbance readings were recorded at 540 nm by using a UV–vis spectrophotometer (FEMTO, 800XI, Brazil).

### 2.3 Characterization of Biomass via Lignocellulosic Analysis

Samples were characterized for ash,^26^ moisture,^27^ volatiles, extractives,^28^ holocellulose, α-cellulose, and hemicellulose contents following the lignocellulosic analysis procedure previously described,^29^ with adaptations.

For ash analysis, approximately 2 g of each sample was calcined at 600 °C in a muffle furnace (GP Científica, GP 2000F, Brazil) for 4 h, followed by a gradual reduction to 200 °C over 1 h. The results are expressed as the ash content (AC%). Moisture and volatile contents were determined by drying approximately 6 g of each sample in an oven (DeLeo, DL-SE05, Brazil) at 105 °C until a constant weight was achieved, and the results were expressed as the moisture content (MC%).

Extractives were determined using a Soxhlet extraction (Nova Ética, 211-6, Brazil) with an acetone-ethanol (2:1) mixture for 8 h. The cellulose cartridges were preserved, and the flask/extractives sets were subjected to rotary evaporation (Fisatom, 801, Brazil) at 60 °C for 15 min and dried in an oven (DeLeo, DL-SE05, Brazil) at 90 °C for 1 h to remove the solvent. The results are expressed as the extractive content (EC%).

Holocellulose was quantified by treating approximately 5 g of extractive-free biomass with sodium chlorite (NaClO_2_) and glacial acetic acid at 70 °C under mild agitation, followed by filtration, acetone washing, and oven (DeLeo, DL-SE05, Brazil) drying at 105 °C for 2 h. The results are expressed as the holocellulose content (HC%). α-Cellulose was isolated from holocellulose using 17.5% sodium hydroxide (NaOH), maceration with a pestle, filtration, and oven (DeLeo, DL-SE05, Brazil) drying at 105 °C for 1 h, with results expressed as α-cellulose content (αC%). The hemicellulose (HeC%) content was calculated as the difference between the holocellulose and α-cellulose percentages relative to the initial sample fraction.

### 2.4 Ionic Liquid Synthesis

The synthesis of the triethylammonium hydrogen sulfate IL was conducted using the method described by Gschwend et al.^15^ A 500 mmol sulfuric acid solution was added dropwise to triethylamine in a round-bottomed flask under magnetic stirring in an ice bath. The mixture was stirred at room temperature for 2 h after the acid addition, followed by rotary evaporation (Fisatom, 801, Brazil) at 60 °C. The synthesized compound was analyzed for pH and infrared (IR) spectra (PerkinElmer, Frontier) at the Analytical Center of the Federal University of Technology, Paraná (UTFPR), Pato Branco Campus.

### 2.5 Application of Biomass Pretreatments Using Ionic Liquids

Two pretreatment methods using the produced IL were applied on BP, OP, and MP samples based on Wei et al.^30^ and Anuchi, Campbell, and Hallett.^31^ In both methods, 1 g of biomass was mixed with 5 g of the IL. For the Wei et al.^30^ method, samples were heated in an oil bath at 100 °C for 2 h, cooled, washed with ethanol and water, and centrifuged to separate the solid fraction, which was dried in an oven (DeLeo, DL-SE05, Brazil) at 55 °C overnight, yielding cellulose pulp. The liquid fraction was rotary evaporated (Fisatom, 801, Brazil) at 70 °C for 2 h to concentrate the IL, which was mixed with washing water from the pulp, and stirred at room temperature for 30 min. Subsequently, the mixture was centrifuged, producing a liquid phase (reserved), and a solid phase dried at 42 °C overnight, representing the precipitated lignin.

For the method proposed by Anuchi, Campbell, and Hallett,^31^ samples were heated in an oven (SolidSteel, SSDi-11L, Brazil) at 100 °C for 1 h, cooled to room temperature for 1 h, washed with ethanol, centrifuged, and Soxhlet extracted (Nova Ética, 211-6, Brazil) for 8 h. The solid phase was dried at room temperature for 24 h, resulting in a cellulose pulp. The reserved liquid phase was rotary evaporated (Fisatom, 801, Brazil) at 70 °C for 2 h. Ultrapure water (15 mL) was added to the concentrated liquid at a ratio of 3 mL per 1 g of IL, the mixture was centrifuged, and the process was repeated twice. Finally, the liquid and solid phases were separated, and the solid was dried overnight in an oven (DeLeo, DL-SE05, Brazil) at 45 °C, yielding precipitated lignin.

For the cellulose pulps obtained by both methods, scanning electron microscopy (SEM) (Zeiss, EVO MA 15, Germany) analyses were performed at the Multi-User Materials Characterization Center of the Federal University of Technology, Paraná, Curitiba campus.

In both methods, the final step involved recovering the IL by rotary evaporation (Fisatom, 801, Brazil) of the liquid phase, resulting from lignin precipitation at 60 °C, to remove water. The recovered IL was analyzed via IR spectroscopy (PerkinElmer, Frontier) at the Analytical Center of the Federal University of Technology, Paraná, Pato Branco campus, for comparison with the synthesized IL before application.

## 3 Results and Discussions

### 3.1 Raw Material Characterization

The results obtained for moisture and volatile contents can be observed in Table 1.

**Table 1:** Moisture and Volatile Content by Drying

residue	moisture and volatile content (%)
Banana peels (BP)	83.40
Orange peels (OP)	76.46
Mango peels (MP)	83.62

Rodrigues et al.^32^ determined a moisture content of approximately 90% for green bananas (*Musa* spp. cv. Prata) peels. Whereas Gbiete et al.^33^ reported a value of 88.11% for BP of the species *Musa acuminata* Cola. Moisture values are dependent on the climate as well as storage conditions of fruits after harvesting. For OP, Ayala et al.^34^ reported a moisture content of 73.53, whereas Ortiz-Sanchez et al.^35^ for OP waste obtained 78.53% of moisture content. For MP, Kučuk et al.^36^ determined values of 77.74% moisture. Mango peels from Palmer, Haden, Keitt, and Espada Vermelha varieties were compared in terms of moisture content by Troiani et al.^37^ which observed values approximately 74–77%, with the highest presented by the variety Espada. Variations with the values reported in the literature are expected since moisture values are dependent on climate as well as storage conditions of fruits after harvesting.

Using the DNS method for fresh biomass samples and their respective dried biomass, the calibration curve (eq 1) was obtained with *R* ^2^ = 0.9998, enabling the calculation of sugar concentrations present in the samples.

1 $$\mathrm{concentration}=2.4983^{*}\mathrm{absorbance}$$

Table 2 presents the total sugar concentration data for the samples, expressed as the means of triplicate measurements with the calculated standard deviation.

**Table 2:** Reducing Sugar Concentration in the Samples

residue	reducing sugar concentration (%)
Banana peels (BP)	8.827 ± 0.001
Orange peels (OP)	11.659 ± 0.001
Mango peels (MP)	16.322 ± 0.002

Reducing sugar is a substrate used by microorganisms, transforming it into alcohol and other byproducts.^38^ Mango residues exhibited the highest sugar concentrations, highlighting their potential as a promising feedstock for ethanol production.^39–41^ For ripe banana peel flour, Salih et al.^42^ determined the value 15%. Regarding dried Orange Peels, Rivas et al.^43^ determined 16.9%, reflecting methodological differences. For dry MP, Lebaka et al.^44^ reported 20.00%, whereas Fronteras et al.^45^ reported a value of 18.65% for total sugars. Wongkaew et al.^46^ found values of 29.00 and 31.57% for xylose and glucose, respectively. In general, the results are consistent with those reported in the literature with variations attributed to the differences in drying methodologies and processes.

Furthermore, analyses of the ash content (AC%), moisture (MC%), extractives (EC%), holocellulose (HC%), α-cellulose (αC%), and hemicellulose (HeC%) were conducted, and the results are summarized in Table 3.

**Table 3:** Results of the Lignocellulosic Analysis of Raw Materials

analysis/residue	Banana peels (BP)	Orange peels (OP)	Mango peels (MP)
AC%	11.046 ± 0.094	3.260 ± 0.029	2.720 ± 0.029
MC%	11.020 ± 0.176	12.577 ± 0.312	12.662 ± 0.056
EC%	19.364 ± 2.011	27.639 ± 8.378	20.237 ± 2.293
HC%	24.459 ± 1.239	28.672 ± 3.544	18.285 ± 0.590
αC%	14.004 ± 0.287	13.539 ± 2.324	10.603 ± 0.303
HeC%	10.456 ± 1.043	15.133 ± 4.971	7.682 ± 0.413

Biomasses with high ash and moisture contents compromise combustion, reducing the fixed carbon content and calorific value while increasing the energy costs of the process.^47^ Among the samples analyzed, the BP the results for ashes obtained in this study are consistent with those reported by Dewi et al.^48^ that reported results for ripe banana peels of three varieties that were between 9.47, 12.73, and 14.24%, but differed from the results of Salih et al.,^42^ who reported an ash content of 0.3%, probably due to different methods of analysis. For OP, Rivas et al.^43^ reported values of 3.5%, very close to those found in this study, and Almowallad et al.^49^ reported values of 4.3%. For MP flour, Fronteras et al.^45^ found an ash content of 0.67%. Wongkaew et al.^46^ reported ash values of around 0.54%. The ash values for the cited authors are lower than what we found, which is probably due to the different methods used for the analysis as well as the varieties used.

The moisture content values of 12% for the BP and OP samples are consistent with those reported in the literature.^48,50,43^ For the mango peels, the results are in accordance with those described in the literature, as Hussain et al.^51^ reported 5.16% and Rocha et al.^52^ reported moisture values of approximately 9%, while discrepancies for MP were observed due to methodological differences. Regarding extractive analysis, the variations between the results of this study and those in literature can be attributed to differences in the solvents and extraction times employed.^53,54^

The compositions of holocellulose, α-cellulose, and hemicellulose determined in this study align with those reported by Alzate Acevedo et al.^55^ and Mishra et al.^56^ for banana and Bicu and Mustata^57^ for orange, presenting divergences for MP when compared to Banerjee et al.^58^

Physicochemical characterization analyses indicated that mango biomass stands out because of its high fermentable sugar content, favorable cellulosic composition for pretreatment processes, low ash content, and moderate moisture levels. Therefore, it is the most promising biomass analyzed for the ethanol production process and its efficiency as a biofuel.

### 3.2 Triethylammonium Hydrogen Sulfate

To characterize the synthesized ionic liquid, its pH was determined, resulting in an acidic ionic liquid with a pH of 4. This result is consistent with that of Zahari et al.,^59,59^ who reported low pH value for the same IL.

Additionally, IR analysis and H^1^NMR of the IL was performed, as shown in Figures S2 and S3, respectively, Supporting Information (SI). This analysis enabled the identification of characteristic bands corresponding to each chemical functional group involved in the synthesis reaction.^60^ In Figure S2 (SI), bands corresponding to the functional groups present in the structure of triethylammonium hydrogen sulfate are observed. The following functional groups were identified: hydroxyl (–OH), around 3500 and 3400 cm^–1^; alkanes (–CH_2_ and –CH_3_), present in bands at wave numbers of 1500 and 1400 cm^–1^, respectively; amine (–NH) between 1640 and 1560 cm^–1^; and sulfone (–S=O), found between 1200 and 1000 cm^–1^. The H^1^NMR spectrum (Figure S3, SI) shows the following peaks: 1.0 ppm, *t*. 9H­(–CH_3_) and 2,9 ppm, *q* 6H (–CH_2_–). In 4.7 ppm, it is possible to see a peak corresponding to the water presence. The δ values are in accordance with the literature.^59^

### 3.3 Cellulose Pulp

Cellulose, a polysaccharide that makes up part of cellular plants, is the most abundant organic polymer in the world, but it is insoluble in water and almost all organic solvents. Currently, cellulose is an important source of biofuels, as well as other chemical compounds, and it is under research to deconstruct its structure into smaller sugars. First, it is necessary to isolate them from cellular plants. TEA is a protic ionic liquid with several attributes: low cost, easy synthesis and storage, Brønsted acid that can deconstruct various biomasses, and lignin dissolution.^59^

The results obtained for the cellulose pulp content for both pretreatments are presented in Table 4.

**Table 4:** Cellulose Pulp Content Resulting from the Pretreatments

sample/methodology	Oil bath (%)	Oven (%)
Banana peels (BP)	40.196 ± 4.734	45.344 ± 0.273
Orange peels (OP)	36.398 ± 5.851	35.263 ± 0.637
Mango peels (MP)	25.123 ± 0.774	27.817 ± 2.438

Analysis of the data in Table 4 indicates that the oven pretreatment proposed by Anuchi, Campbell, and Hallett^31^ provided better reaction conditions, resulting in cellulose pulp values with lower deviations when compared with the oil bath methodology proposed by Wei et al.^30^ Regarding biomass, BP stands out for exhibiting the highest cellulose pulp production, indicating its potential for the hydrolysis step, providing larger amounts of cellulose for conversion into sugars. However, the results obtained may have been influenced by the presence of residual ionic liquid and lignin, which were not completely removed during washing.

Furthermore, SEM analyses were performed at a magnification of 5,000 x, as shown in Figure 2, to observe whether there were any structural changes in the biomass after pretreatment with the IL.

**Figure 2:**
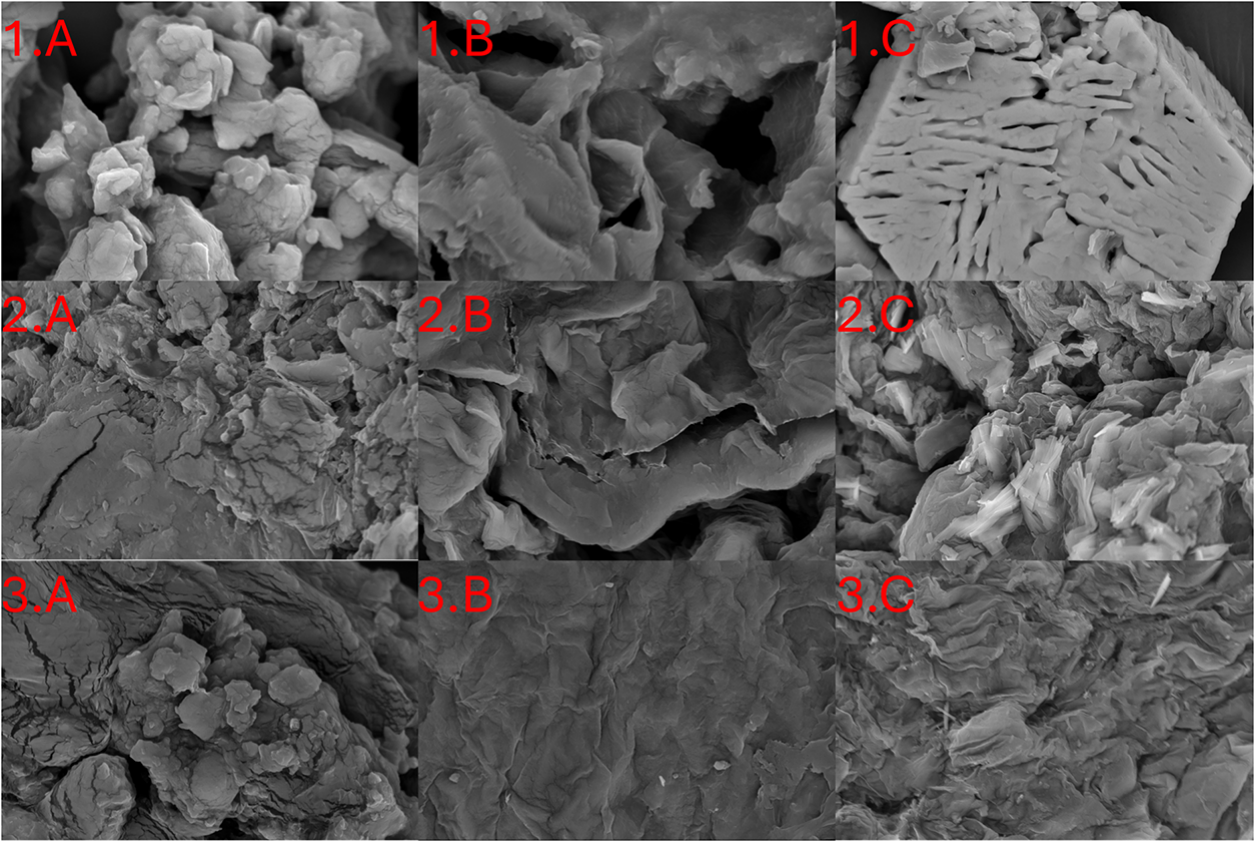
SEM analysis for (1) BP, (2) OP, and (3) MP, showing (A) without pretreatment and with pretreatment in (B) oil bath and (C) oven.

Analysis of the results from the SEM examination revealed that the biomasses exhibited heterogeneity, which was characterized by particles of diverse dimensions and morphologies. Moreover, structural modifications were evident in biomass samples before and after the application of pretreatments.^56,61^ Furthermore, micrographs obtained from the oven pretreatment methodology demonstrated a more pronounced formation of fragmented particles, suggesting enhanced efficacy in the disruption of original structures and lignin-hemicellulose-cellulose complexes.

### 3.4 Precipitation of Insoluble Lignin

Table 5 shows the results of the precipitated lignin content during the pretreatments.

**Table 5:** Insoluble Precipitated Lignin Contents

lignin/methodology	Oil bath (%)	Oven (%)
Banana peels (BP)	4.834 ± 2.908	4.386 ± 0.616
Orange peels (OP)	4.036 ± 1.834	3.747 ± 0.377
Mango peels (MP)	3.499 ± 2.015	4.558 ± 0.933

Owing to potential experimental errors related to temperature control during the process, the oil bath methodology exhibited high standard deviations. Conversely, the oven method demonstrated greater reliability, proving to be effective in lignin precipitation, particularly for MP residues. This methodology indicates that MP is more susceptible to pretreatment at 100 °C, yielding a higher lignin removal efficiency.

Given this efficiency in lignin removal from mango, the previously observed low cellulose pulp content is substantiated. Furthermore, a reduction in impurities and greater potential for hydrolysis processes were indicated, thereby enhancing the production of reducing sugars for ethanol production during fermentation.

Hasanov, Raud and Kikas^62^ describe the use of various ionic liquids for the process of separating lignin from lignocellulosic material, all of which are derived from imidazolium, pyridine, pyrrolidine, amine and posfonium cations combined with anions such as BF_4_^–^, FAP-, Tf_2_N^–^, OTF^–^, Cl^–^, PF_6_^–^, CH3COO^–^, and N­(CN)_2_^–^ which makes them expensive for industrial use. The same authors reported that protic ionic liquids derived from the transfer of a proton between a Brønsted acid and a Brønsted base have become more efficient and cheaper, with characteristics that can dissolve lignin, which corroborates the results of this study.

### 3.5 Recovery of Ionic Liquid

Subsequently, in both pretreatment methodologies, the recovery step for the ionic liquid triethylammonium hydrogen sulfate employed in the processes was conducted. Table 6 presents the recovered ionic liquid contents.

**Table 6:** Recovered Ionic Liquid Content

ionic liquid/methodology	Oil bath (%)	Oven (%)
Banana peels (BP)	82.914 ± 12.217	89.333 ± 1.929
Orange peels (OP)	60.074 ± 17.807	95.229 ± 2.519
Mango peels (MP)	74.584 ± 19.304	96.344 ± 3.829

It should be noted that for the oil bath methodology, the standard deviations increased owing to experimental errors, as mentioned in the previous section.

Upon analysis of the data obtained, it is evident that oven pretreatment yielded more significant and consistent results in the recovery of the IL. This recovery is crucial for the economic viability of the process as it enables the reuse of the IL within the pretreatment process itself, thereby eliminating the need for additional synthesis and consequently reducing the process cost.

Due to the high standard deviations in the results presented for the Wei et al.^30^ method, the discussion regarding which residue achieved the optimal recovery was inconclusive. However, analysis of the results from the Anuchi, Campbell, and Hallett^31^ method suggests comparable outcomes, indicating that the efficiency of the ionic liquid recovery step is not influenced by the biomass utilized. Furthermore, infrared (IR) spectroscopic analyses were conducted to evaluate the compounds present in the recovered ionic liquids (ILs), based on the analysis of the pure IL presented in Figure S2 (SI). Figures S4–S6 (SI) illustrate a comparison of the IR spectra of the ILs recovered in the pretreatments for the fruit peels.

Performing the analysis according to Silverstein, Webster, and Kiemle,^60^ the functional groups present in the structure of triethylammonium hydrogen sulfate were identified, matching the analysis performed for pure IL. The recovered ILs may contain sugars, such as glucose and fructose, as evidenced by the aldehyde and carbonyl bands, although identification through it may have been hindered by interference from other bands. Therefore, it is essential to separate and recover these sugars, enabling fermentation and reuse of the purified ionic liquid.

## Conclusions

The characterization of residues of banana, orange, and mango peels demonstrated the potential source of lignocellulosic biomasses. Among these, mango exhibits superior characteristics, presenting high levels of fermentable sugars, low ash and moisture contents, and favorable cellulosic composition for the pretreatment and hydrolysis steps. As for the pretreatments studied, the oven-based method proved to be efficacious, particularly for mango peels, promoting greater lignin removal and precipitation, resulting in low levels of impurities in the cellulose pulp. The recovery of triethylammonium hydrogen sulfate is crucial for the feasibility of the process, demonstrating consistent results regardless of the biomass utilized. Infrared analysis confirmed the presence of the original ionic liquid (IL), despite potential interference from the diluted sugars. In conclusion, mango peels represent the most promising biomass source for this process. However, technical and economic challenges remain to be overcome to enable commercial-scale application, necessitating further studies to improve the efficiency and sustainability of IL-based pretreatment processes for 2G ethanol production.

## Supplementary Material



## Notes

Reaction of the TEAH and characterization by infrared (IR) and proton nuclear magnetic resonance (^1^H NMR) spectroscopy; IR analysis of the ionic liquids recovered in the pretreatments from oil bath and oven for BP, OP, and MP (PDF)
