# Supplementary material for: Green Pretreatment of Tropical Fruit Peels Using Triethylammonium Hydrogen Sulfate: A Route Toward Sustainable Biomass Valorization
Source: ACS Omega. 2026 Feb 2;11(6):9696–703. doi: 10.1021/acsomega.5c10185 (PMC12917712; doi:10.1021/acsomega.5c10185)
Supplement: Supplementary file 1 [file ao5c10185_si_001.pdf]

Supplementary Information

"Green Pretreatment of Tropical Fruit Peels Using Triethylammonium Hydrogen Sulfate: A Route Toward Sustainable Biomass Valorization"

Leonardo A. F. Souza[1], Crystian Ribas[2], Irede Dalmolin[2], Marcelo Bortoli[2], Tania Maria Cassol[2]

[1]Universidade Estadual de Campinas (Unicamp), School of Chemical Engineering (FEQ), Av. Albert Einstein, 500, Cidade Universitária, Campinas, SP, ZIP Code 13083-852, Brazil.

[2]Universidade Tecnológica Federal do Paraná (UTFPR), R. Gelindo João Folador, 2000, B. Novo Horizonte, Francisco Beltrão, PR, ZIP Code 85602-863, Brazil.

Telephone: 55 46 991202601

1. Reaction and characterization of TEAH by infrared spectroscopy.

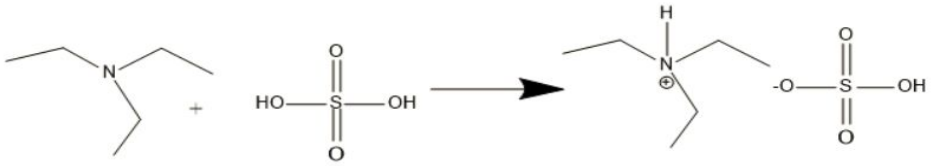

(1)

Figure S1 – Acid-base reaction for the formation of triethylammonium hydrogen sulfate

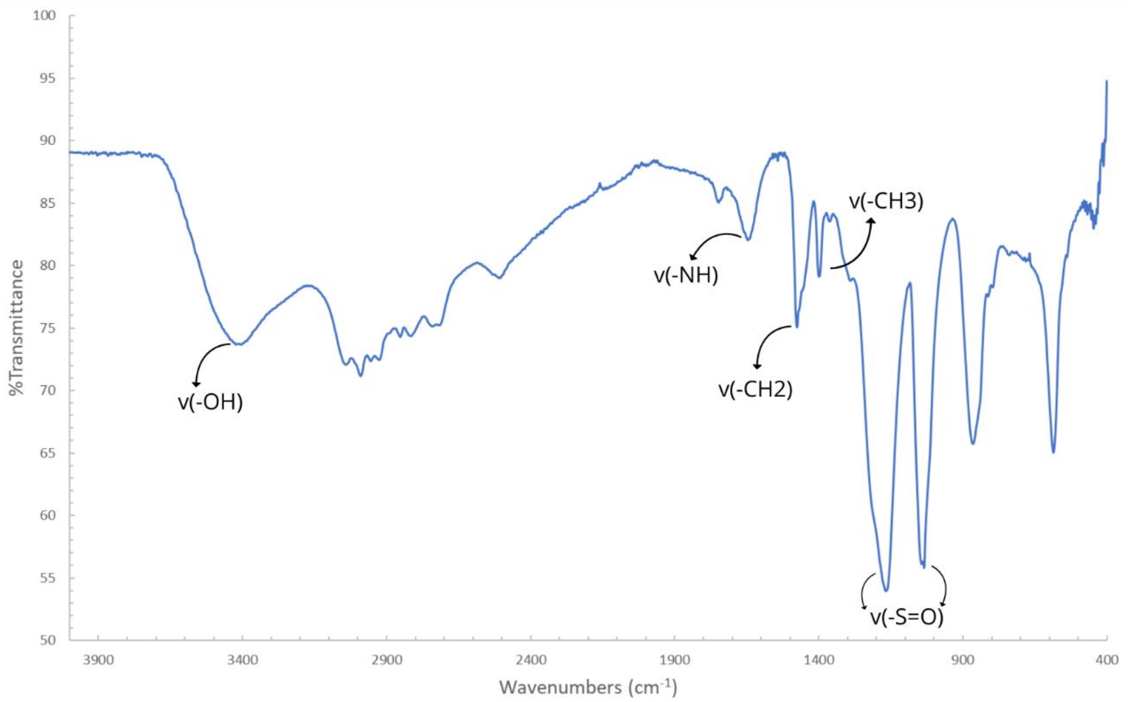

Figure S2 – Infrared (IR) analysis of triethylammonium hydrogen sulfate

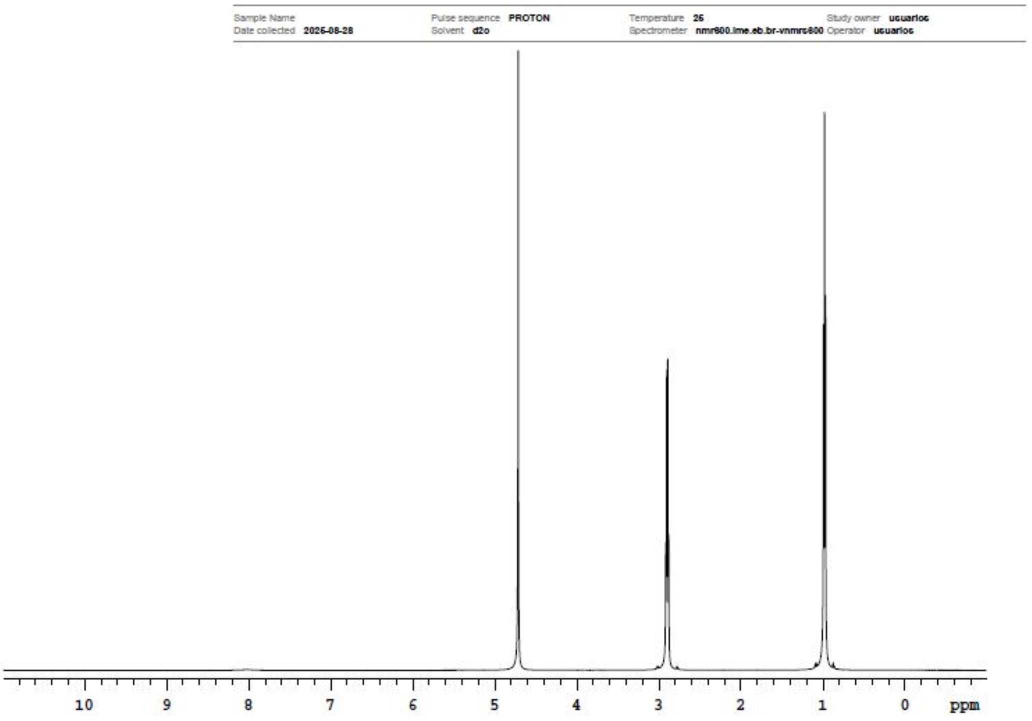

Figure S3 – Proton Nuclear Magnetic Resonance (H^1NMR) spectroscopy of triethylammonium hydrogen sulfate

2. Recovery of the ionic liquid

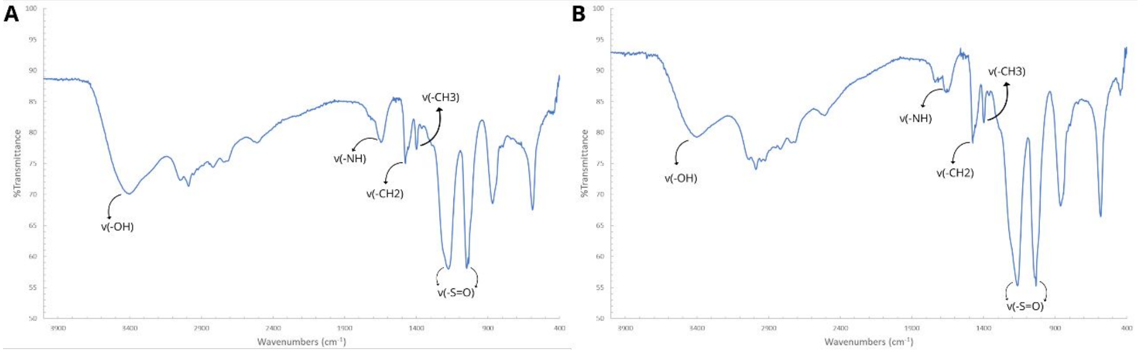

Figure S4 – Infrared (IR) analysis of the ionic liquids recovered in the pretreatments from (A) oil bath and (B) oven for BP

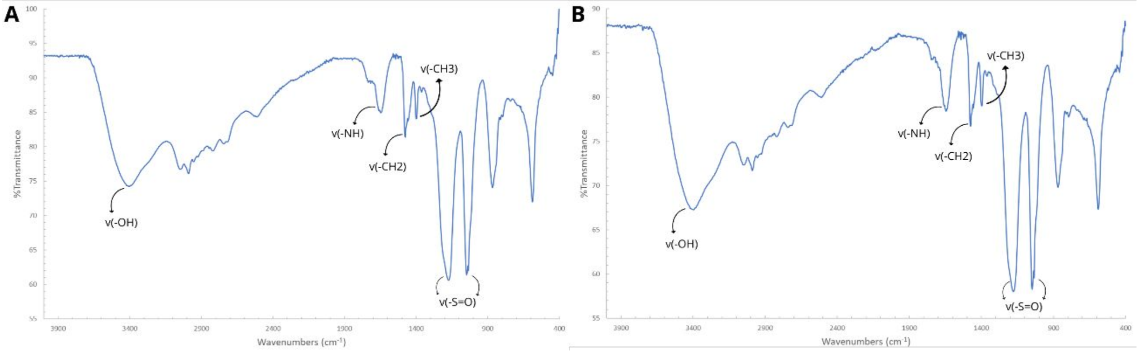

Figure S5 – Infrared (IR) analysis of the ionic liquids recovered in the pretreatments from (A) oil bath and (B) oven for OP.

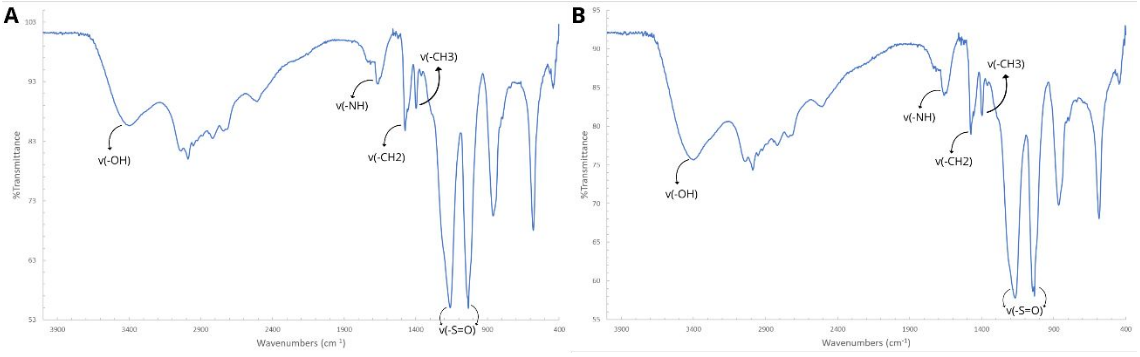

Figure S6 – Infrared (IR) analysis of the ionic liquids recovered in the pretreatments from (A) oil bath and (B) oven for MP.
